# Association between clinical variables related to asthma in schoolchildren born with very low birth weight with and without bronchopulmonary dysplasia

**DOI:** 10.1016/j.rppede.2016.03.005

**Published:** 2016

**Authors:** Emília da Silva Gonçalves, Francisco Mezzacappa-Filho, Silvana Dalge Severino, Maria Ângela Gonçalves de Oliveira Ribeiro, Fernando Augusto de Lima Marson, Andre Moreno Morcilo, Adyléia Aparecida Dalbo Contrera Toro, José Dirceu Ribeiro

**Affiliations:** a Departamento de Pediatria, Faculdade de Ciências Médicas, Universidade Estadual de Campinas, Campinas, SP, Brazil

**Keywords:** Asthma, Bronchopulmonary dysplasia, Preterm birth

## Abstract

**Objective:**

To assess the prevalence, spirometry findings and risk factors for asthma in schoolchildren who were very low birth weight infants with and without bronchopulmonary dysplasia.

**Methods:**

Observational and cross-sectional study. The parents and/or tutors answered the International Study of Asthma and Allergies in Childhood questionnaire. The schoolchildren were submitted to the skin prick test and spirometry assessment.

**Results:**

54 schoolchildren who were very low birth weight infants were assessed and 43 met the criteria for spirometry. Age at the assessment (bronchopulmonary dysplasia=9.5±0.85; without bronchopulmonary dysplasia=10.1±0.86 years) and birth weight (bronchopulmonary dysplasia=916.7±251.2; without bronchopulmonary dysplasia=1171.3±190.5g) were lower in the group with bronchopulmonary dysplasia (*p*<0.05). The prevalence of asthma among very low birth weight infants was 17/54 (31.5%), being 6/18 (33.3%) in the group with bronchopulmonary dysplasia. There was an association between wool blanket use in the first year of life (*p*=0.026) with the presence of asthma at school age. The skin prick test was positive in 13/17 (76.5%) and 23/37 (62.2%) of patients with and without asthma, respectively. The schoolchildren with asthma had lower *z*-score values of forced expiratory flow between 25% and 75% of forced vital capacity (*n*=16;−1.04±1.19) when compared to the group of patients without asthma (*n*=27;−0.380.93) (*p*=0.049). There was no difference between the spirometry variables in the groups regarding the presence or absence of bronchopulmonary dysplasia.

**Conclusions:**

Very low birth weight infants with and without bronchopulmonary dysplasia showed a high prevalence of asthma (33.3% and 30.6%, respectively). Pulmonary flow in the small airways was lower in children with asthma.

## Resumo

**Objetivo:**

Avaliar prevalência, espirometria e fatores de risco para asma em escolares que foram recém-nascidos de muito baixo peso com e sem displasia broncopulmonar.

**Métodos:**

Estudo observacional e transversal. Aplicou-se aos pais e/ou responsáveis o questionário International Study of Asthma and Allergies in Childhood. Foi feito teste cutâneo de hipersensibilidade imediata e espirometria nos escolares.

**Resultados:**

Avaliados 54 escolares que foram recém-nascidos de muito baixo peso e 43 preencheram critérios para espirometria. A idade na avaliação (displasia broncopulmonar=9,5±0,85; sem displasia broncopulmonar=10,1±0,86 anos) e o peso de nascimento (displasia broncopulmonar=916,7±251,2; sem displasia broncopulmonar=1.171,3±190,5 g) foram menores no grupo com displasia broncopulmonar (*p*<0,05). A prevalência de asma entre os recém-nascidos de muito baixo peso foi de 17/54 (31,5%); no grupo com displasia broncopulmonar, de 6/18 (33,3%). Houve associação entre uso de cobertor de lã no primeiro ano de vida (*p*=0,026) com presença de asma na idade escolar. O teste cutâneo de hipersensibilidade imediata foi positivo em 13/17 (76,5%) e 23/37 (62,2%) nos grupos com e sem asma, respectivamente. Os escolares com asma apresentaram menores valores em z-score do fluxo expiratório forçado entre 25% e 75% da capacidade vital forçada (n=16; −1,04±1,19) comparados com os do grupo de pacientes sem asma (n=27; −0,38±0,93) (*p*=0,049). Não houve diferença entre as variáveis da espirometria no grupo com relação à presença ou não de displasia broncopulmonar.

**Conclusões:**

Os recém-nascidos de muito baixo peso, com e sem displasia broncopulmonar, apresentaram prevalência elevada de asma (33,3% e 30,6%, respectivamente). Os fluxos pulmonares das pequenas vias aéreas foram menores nos escolares com asma.

## Introduction

Since 1960, with the improvement of perinatal care, there has been a reduction in mortality of newborns (NB) with birth weight (BW) of less than 1500g, termed very low birth weight (VLBW) infants.^1^ In contrast, there has been an increase of complications of prematurity, the most serious of which is bronchopulmonary dysplasia (BPD), the earliest chronic obstructive pulmonary disease that affects humans.^1^

During this period the prevalence of BPD is high, and it increases in lower gestational ages and smaller infants.^2^ BPD affects 30% of VLBW infants<1000g, 52% of those weighing between 501 and 750g, and 7% of those weighing between 1251 and 500g.^2^^,^ 3

The manifestations of prematurity include immune imbalance, increased susceptibility to viral infections,^4^^,^ 5 and allergen absorption.^6^ Infants with BPD present chronic lung inflammation, which predisposes them to infections and hospitalizations. These risk factors may be associated with the prevalence of wheezing in infants^2^ and asthma in childhood and adolescence.^7^^-^ 14

Children with BPD have a high prevalence of recurrent wheezing.^6^ In contrast, the effects of BPD in the onset of atopy, asthma, and/or recurrent wheezing at older ages is controversial.^3^^,^ 4 ^,^ 9 ^,^ 13 ^,^ 15 ^,^ 16 The effect of BPD in lung function in the long term has been evaluated in numerous age groups, including newborns,^17^ infants,^18^ preschoolers,^19^ schoolchildren,^7^^-^ 11 adolescents,^12^^-^ 14 and adults.^20^^-^ 22 Studies have shown reduced lung volumes in different age groups, but with low association to clinical symptoms. This fact has not been explained in the literature.

Asthma is a disease of great importance in global public health.^23^ The prevalence ranges from 2.1% to 32.2% and from 4.1% to 32.1% in children and adolescents, respectively.^23^ However, studies have not been able to determine which is the main risk factor for childhood asthma in VLBW infants, whether prematurity itself or BPD.

Considering that preterm infants have been increasingly presenting a favorable outcome and that there are few studies in Brazil on asthma prevalence in preterm infants with and without BPD,^7^^,^ 22 this study aimed to determine the presence of asthma and atopy in children born with VLBW at school age and to assess lung function by spirometry, according to the presence or absence of BPD.

## Method

This was an observational and cross-sectional study approved by the Institutional Review Board of the University Hospital under No. 569/2010 and No. 605/2010. All parents and/or guardians signed an informed consent. Initially, all records of VLBW infants born between October 2000 and November 2004 at the university hospital were selected. Deaths and cases with chronic diseases, genetic diseases, vascular ring, diaphragmatic hernia, pulmonary sequestration, chest wall deformity, and ciliary dyskinesia were excluded.

The diagnosis of BPD was established in infants who, after 28 days of life, had respiratory failure and depended on oxygen at over 21% concentration to maintain partial pressure of oxygen>50mmHg.^1^ Two groups were considered: BPD and no BPD.

Telephone calls were made to invite schoolchildren aged between 7 and 12 years to participate in the study. The aforementioned exclusion criteria were again applied. A personal interview was conducted with parents and/or guardians, who answered the written International Study of Asthma and Allergies in Childhood (ISAAC) Phase I (IQ) and complementary ISAAC phase II (CIQ) questionnaires.^23^ The risk factors assessed by CIQ^23^ regarding the first year of life and the past year were: breastfeeding duration; older siblings (regardless of the presence of atopy and/or asthma); age at start of daycare or primary school attendance; paternal and maternal atopy; contact with animals inside and outside the home, current smoking at home; current, prenatal, and postnatal maternal smoking; presence of mold on the walls; type of floor, pillow, and blanket; and residence in rural or urban area. Questions about family history of atopy in siblings, parents, and grandparents; pneumonia; atopic dermatitis; allergic rhinitis; bronchiolitis; heart disease; hospitalizations; drug allergies; and relief and routine asthma medication were also asked.

Asthma was defined as positive answers to question 2 of the IQ: “In the last 12 (twelve) months, has your child had wheezing?” This question has a sensitivity of 100%, specificity of 78%, positive predictive value of 73%, and negative predictive value of 100% for the diagnosis of asthma.^23^ The prevalence of severe asthma was considered in those who answered “yes” to at least two of the following IQ questions:^23^ “in the last 12 (twelve) months, how many wheezing crises has your child had?”; “In the last 12 (twelve) months, how often was your child's sleep disturbed by wheezing?”; “In the last twelve (12) months, was the hiss of your child so strong as to prevent him/her from saying more than two words between every breath?”; or “In the last 12 (twelve) months, has your child had wheezing after exercise?”

The participants underwent clinical examination, skin prick test (SPT), spirometry, and measurement of bronchial hyperresponsiveness (BHR) through a concentration of methacholine that produced a 20% decrease in the forced expiratory volume in one second (FEV_1_) at spirometry (PC20 methacholine). All procedures were performed in lung physiology laboratory of the Pediatrics Research Center, School of Medical Sciences.

The SPT was performed by puncture^23^ technique on the forearm with plastic lancets (Alergoprick-Flexor™, Sertãozinho, SP, Brazil). A set of lancets was used for each child, one for each extract. The following purified glycerinated extracts at 50%, manufactured by IPI-ASAC™ (São Paulo, SP, Brazil), were used: *Dermatophagoides pteronyssinus*, *D. farinae*, *Blomia tropicalis*, *Blatella germanica*, fungus mix (*Alternaria alternata*, *Cladosporodium herbarum*, *Aspergilus fumigatus* and *Penicilium*), *Canis familiaris*, *Felis domesticus.* The following controls were used: positive (histamine) and negative (0.9% saline solution). The results were measured after 15min; a reaction was considered positive if wheal size ≥3mm, with non-reactive negative control and positive control with wheal size ≥3mm.^23^ The presence of a positive response in the SPT was considered as atopy.

Spirometry assessed the forced vital capacity (FVC), FEV_1_, Tiffeneau index (FEV_1_/FVC), and mean expiratory flow between 25% and 75% of FVC (FEF 25-75%). A CPFS/D model spirometer (Medical-Graphics, Saint Paul, Minnesota, United States) was used. Spirometry followed the recommendations of the European Respiratory Society (ERS) and the American Thoracic Society (ATS).^24^ The child took the test while standing and used the nose clip with open maneuver. The child was requested to perform a vigorous and prolonged expiratory maneuver to achieve the reproducibility criteria of the spirometry test software: Breeze PF version 3.8 for Windows 95/98/NT. The evaluation of FVC, FEV_1_, FEV_1_/FVC, and FEF 25-75% was conducted according to the standard reference equations by Quanjer et al.^24^ based on age, sex, weight, and ethnicity. Airway obstruction was described as FEV_1_/FVC ratios below the lower limit of normal; for severity, the FEV_1_% *z*-score was used in two categories: (i) mild obstruction: *z*-score≥−2 or 70% of the predicted value; (ii) moderate obstruction:−2.5≤*z*-score<−2.0 or 60-69% of the predicted value.^24^^,^ 25

The BHR measurement followed the recommendations of ERS/ATS.^24^ Nebulizations with acetyl-beta-methylcoline (methacoline-chloride; code A2251, Sigma) were made with the following dilutions (in mg/mL): 0.125; 0.25; 0.5; 1; 2; 4; 8; 16; 32. Spirometry was performed at the start of the exam and 1min after each nebulization with methacholine. The exam was interrupted when a ≥20% decrease from baseline FEV_1_ was reached. Then, each subject received inhaled salbutamol (four jets of 100mcg) and after 30min, the last FEV_1_ was recorded. The test was considered positive when a 20% decrease in FEV_1_ occurred with less than 4mg of methacholine, and was graded as (i) mild, at concentrations of 1-4mg/mL methacholine; (ii) moderate to severe, at concentrations<1mg/mL.

Data were processed using SPSS version 16.0 (SPSS Inc., Chicago, United States) and Epi-Info 6.04b (CDC, United States). To compare the ratios, the *χ* ^2^ test was used or, when indicated, Fisher's exact test for 2×2 tables or the Fisher-Freeman-Halton exact distribution test for larger tables. In the case of the Fisher-Freeman-Halton test, bilateral probability was estimated using the Monte Carlo method. To compare the means of two independent groups, Student's *t*-test was used. Prevalences and their 95% confidence intervals were calculated using the exact method (binomial distribution), using the Epitable routine of Epi-Info. The *Odds Ratio* (OR) and its 95% confidence interval for asthma in relation to BPD were determined using the StatCalc routine of Epi-Info. The mean, standard deviation, minimum, and maximum values of the quantitative variables of patients regarding the diagnosis of asthma and BDP were determined. A significance level of *p*<0.05 was adopted.

For the present study, the sample power was calculated using G*Power 3.1.9.2, using as a parameter the group of patients with asthma; at Fisher's exact test, the sample power was>0.8003402, above the cutoff value 80%. The calculation of sample power was made after the study.

## Results

**Figure 1:**
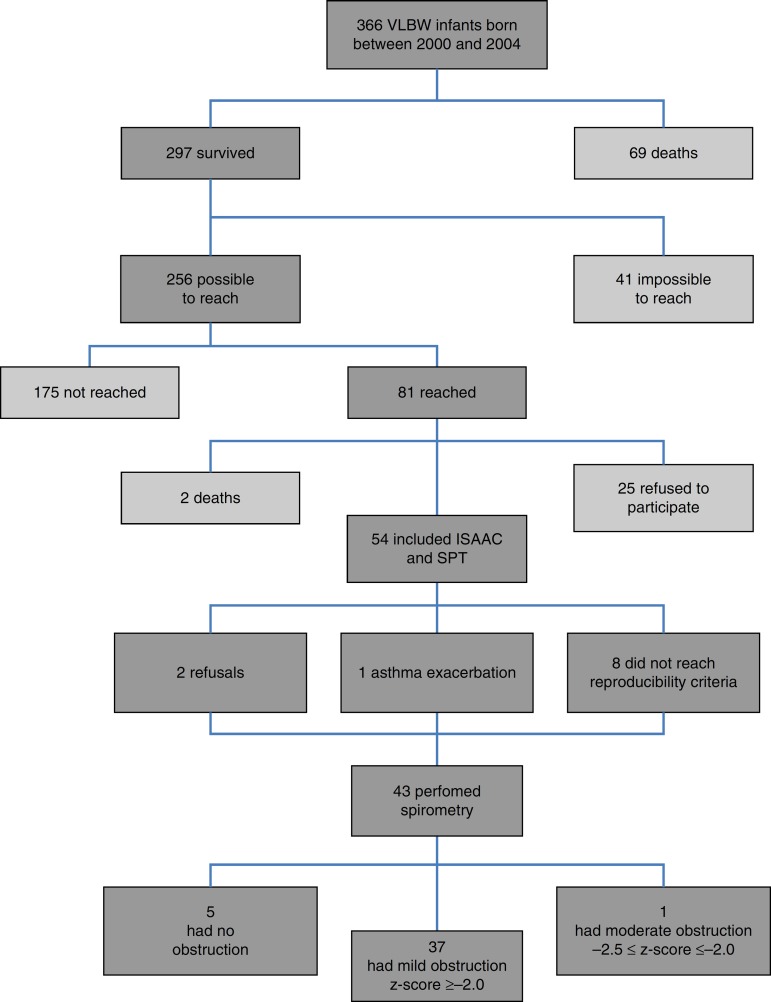
Inclusion flow diagram of very low birth weight (VLBW) infants. ISAAC, International Study of Asthma and Allergies in Childhood; SPT, skin prick test.

Fig. 1 presents the inclusion flow diagram. Of the 54 patients included, 33/54 (61.1%) were male, aged 9.9±0.92 years, and 21/54 (38.9%) female, aged 9.9±0.90 years (*p*=0.810). Male and female VLBW infants had similar BW: 1065.1±243.8g and 1120±243g, respectively (*p*=0.423). There was a higher prevalence of whites (47/54-87%). Table 1 shows the characteristics of VLBW infants with BPD. The age at the time the study was lower in the BPD group (*p*=0.014). Table 2 shows the characteristics of VLBW infants with and without asthma.

**Table 1:** Characterization of the population with birth weight lower than 1500g considering the presence of bronchopulmonary dysplasia (BPD).

Variables^a^	BPD (*n*=18/54)	No BPD (*n*=36/54)	*p*-value	OR (95%CI)
Age at the test (years)^b^	9.5±0.85 (7.9-10.7)	10.1±0.86 (8.2-11.5)	0.14	-
Birth weight (g)^b^	916.7±251.2 (420-1320)	1171.3±190.5 (600-1465)	<0.1	-
Male^c^	13 (72.2%)	20 (55.6%)	0.236	2.052 (0.54-8.97)
White^d^	16 (88.9%)	31 (86.1%)	1.000	1.284 (0.18-14.91)
Presence of asthma^c^	6 (33.3%)	11 (30.6%)	0.836	1.134 (0.27-4.39)
Presence of severe asthma^d^	4 (22.2%)	5 (13.8%)	0.620	2.278 (0.21-35.67)
Prior history of pneumonia^e^				
None	9 (50.0%)	20 (55.6%)	1.000	0.803 (0.25-2.56)
1-3	7 (38.9%)	13 (36.1%)		0.815 (0.07-11.95)
4 or more	2 (11.1%)	3 (8.33%)		1.367 (0.10-13.22)
Positive prick test^c^	10 (55.6%)	26 (72.2%)	0.239	0.488 (0.15-1.64)

BPD, bronchopulmonary dysplasia; g, grams; *n*, number of patients; OR, *Odds Ratio*; 95%CI, 95% confidence interval.

a Data for variables with categorical distribution are represented as number of patients with variable/total number of individuals included in the group and percentage related to the variable analyzed within the possible groups; the data with numerical distribution are presented as mean±standard deviation; minimum-maximum.

b Student's *t*-test.

c *χ* ^2^ test.

d Fisher's exact test.

e Fisher-Freeman-Halton exact test.

**Table 2:** Characterization of the population with birth weight lower than 1500g considering the presence of asthma.

Variables^a^	With asthma (*n*=17/54)	Without asthma (*n*=37/54)	*p*-value	OR (95%CI)
Age at the test (years)^b^	9.7±0.6 (8.5-0.7)	10±1 (7.9-11.5)	0.322	-
Birth weight (g)^b^	1075.4±267.5 (420-1455)	1091.5±234.1 (600-1455)	0.824	-
Male^c^	9 (52.9%)	24 (64.8%)	0.404	0.615 (0.16-2.32)
White^d^	14 (82.3%)	33 (89.1%)	0.809	0.572 (0.08-4.42)
Presence of severe asthma^d^	9 (52.9%)			
Prior history of pneumonia^e^				
None	10 (58.8%)	19 (51.3%)	0.638	1.346 (0.42-4.51)
1-3	5 (29.4%)	15 (40.6%)		0.515 (0.06-5.40)
4 or more	2 (11.7%)	3 (8.1%)		1.499 (0.16-11.06)
Positive prick test^c^	13 (76.5%)	23 (62.16%)	0.364	1.954 (0.473-9.884)

g, grams; *n*, number of patients; OR, *Odds Ratio*; 95%CI, 95% confidence interval.

a Data for variables with categorical distribution are represented as number of patients with variable/total number of individuals included in the group and percentage related to the variable analyzed within the possible groups; data with numerical distribution are presented as mean±standard deviation; minimum-maximum.

b Student's *t*-test.

c *χ* ^2^ test.

d Fisher's exact test.

e Fisher-Freeman-Halton exact test

The prevalence of asthma in VLBW infants was 17/54 (31.5%). The BPD and no BPD groups presented similar prevalence of asthma: 6/18 (33.3%) and 11/36 (30.6%), respectively (*p*=0.836; Tables 1 and 2).

BW in the BPD group (916.7±251.2g) was lower than in the no BPD group (1171.3±190.5g; *p*<0.001). No association between BPD and asthma severity (*p*=0.620) and between BPD and the number of pneumonias (*p*=1.0; Table 1) were observed.

The SPT was positive in 10/18 (55.6%) and 26/36 (72.2%) in the BPD and no BPD groups, respectively (*p*=0.239; Table 1). In the groups with and without asthma, the SPT was positive in 13/17 (76.5%) and 23/37 (62.2%), respectively (*p*=0.364; Table 2). In both cases, a positive association was observed.

Among the variables analyzed for the ISAAC questionnaire, an association was observed with the use of wool blanket in the first year of life (*p*=0.026; [With asthma and wool blanket: 6/17 individuals with asthma] and [without asthma and with wool blanket: 25/37 subjects without asthma]; OR=0.269; 95%CI=0.08-0.088).

**Figure 2:**
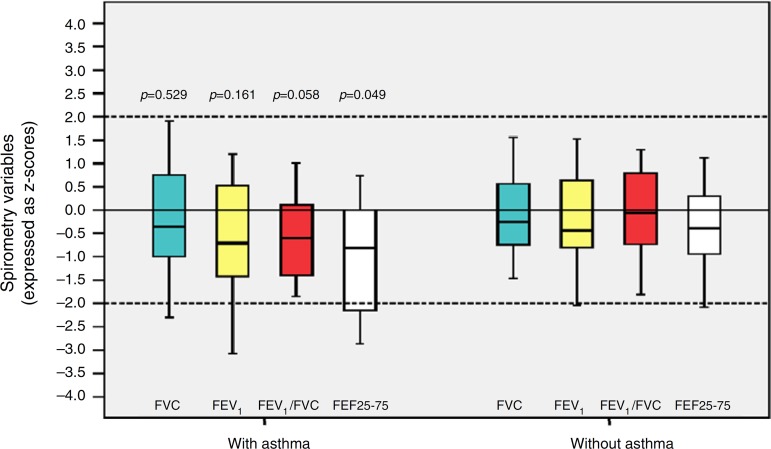
Distribution of *z*-scores in relation to asthma in children and adolescents who were very low birth weight (VLBW) newborns regarding spirometry variables. FVC, forced vital capacity; FEV_1_, forced expiratory volume in one second; FEV_1_/FVC, ratio between FEV_1_ and FVC; FEF 25-75%, mean expiratory flow between 25% and 75% of FVC; p, *p*-value. Medians are present as horizontal lines that divide the rectangles, which contain 50% of values. The bars extend to the maximum and minimum values. Two-tailed Student's *t*-test.

Among the 43 children and adolescents who underwent spirometry, five showed no obstruction, 37 presented mild obstruction, and one teenager presented moderate obstruction.^24^ Table 3 shows the *z*-score distribution of spirometric values in patients with and without BPD compared to those who did or did not develop asthma. The mean *z*-score of FEF 25-75% in patients with asthma (−1.04±1.19) was lower than in the group without asthma (−0.38±0.82; *p*=0.049; Fig. 2). There was no difference between spirometry variables in the BPD and no BPD groups, as well as in the groups with or without asthma.

**Table 3:** Distribution of the *z*-score of spirometry variables (FVC, FEV_1_, FEV_1_/FVC, and FEF 25-75%) of children and adolescents who were very low birth weight (VLBW) infants with and without bronchopulmonary dysplasia (BPD) in relation to those who did or did not develop asthma.

Variables^a^	BPD (12/43) 28%	No BPD (31/43) 72%	*p*-value^b^
*FVC*			
Asthma	-0.37±1.83	-0.26±0.89	0.899
No asthma	+0.21±1.07	-0.21±0.71	0.247

*FEV*_1_			
Asthma	-0.86±2.05	-0.49±1.08	0.641
No asthma	-0.10±1.08	-0.11±0.88	0.978

*FEV*_1_/*FVC*			
Asthma	-0.92±0.90	-0.44±0.95	0.364
No asthma	-0.57±0.76	0.17±0.91	0.067

*FEF25-75%*			
Asthma	-1.38±1.42	-0.89±1.11	0.463
No asthma	-0.71±0.80	-0.27±0.96	0.292
Variables	With asthma (16/43) 37.2%	Without asthma (27/43) 62.8%	*p*-value^b^
*FVC*			
BPD	-0.37±1.83	+0.21±1.07	0.230
No BPD	-0.26±0.89	-0.21±0.71	0.776

*FEV*_1_			
BPD	-0.86±2.05	-0.10±1.08	0.412
No BPD	-0.49±1.08	-0.11±0.88	1.000

*FEV*_1_/*FVC*			
BPD	-0.92±0.90	-0.57±0.76	0.648
No BPD	-0.44±0.95	0.17±0.91	0.066

*FEF25-75%*			
BPD	-1.38±1.42	-0.71±0.80	0.527
No BPD	-0.89±1.11	-0.27±0.96	0.244

FVC, forced vital capacity; BPD, bronchopulmonary dysplasia; FEF 25% -75%, mean expiratory flow between 25% and 75% of FVC; FEV_1_, forced expiratory volume in one second; FEV_1_/FVC, ratio of FEV_1_ and FVC.

a Data for variables with categorical distribution are represented as number of patients with variable/total number of individuals included in the group and percentage related to the variable analyzed within the possible groups; the data with numerical distribution are presented as mean±standard deviation.

b Two-tailed Student's *t*-test.

The values of PC20 methacholine in VLBW group with asthma were lower than in the group without asthma (*p*=0.022). In contrast, the values of PC20 methacholine in the VLBW BPD and no BPD groups were similar (*p*=0.72). Table 4 describes the data regarding the ISAAC variables and the presence of asthma, and Table 5 describes the association of asthma severity with BPD and BW.

**Table 4:** ISAAC risk factors in newborns with very low weight with and without asthma.

Variables^a^		With asthma (17/54) 31.5%	Without asthma (37/54) 68.5%	*p*-value	OR (95%CI)
Breastfeeding duration^b^	>6 months	4 (36.4%)	7 (63.3%)	0.672	1.312 (0.30-5.37)
	<6 months	8 (26.7%)	22 (73.3%)		0.589 (0.14-2.51)
	Absent	5 (38.5%)	8 (61.5)		1.498 (0.38-5.65)
Older siblings^b^	≥4 siblings	3 (42.9%)	4 (57.1%)	0.591	1.748 (0.29-9.55)
	3 siblings	1 (33.3%)	2 (66.6%)		1.18 (0.02-24.73)
	2 siblings	3 (50%)	3 (50%)		2.73 (0.41-18.29)
	1 sibling	2 (16.7%)	10 (83.3%)		0.46 (0.04-2.98)
	No sibling	8 (30.8%)	18 (69.2%)		0.939 (0.29-3.04)
Daycare attendance^b^	<12 months	1 (20%)	4 (80%)	0.416	0.521 (0.01-5.86)
	≥12 months	8 (44.4%)	10 (55.6%)		2.26 (0.56-9.32)
	No	8 (25.8%)	23 (74.2%)		0.547 (0.15-2.02)
Father with asthma^c^	Present	1 (25%)	3 (75%)	1.000	0.891 (0.02-12.41)
Mother with asthma^c^	Present	2 (28.6%)	5 (71.4%)	1.000	0.803 (0.07-5.66)
Parents with asthma^c^	Present	0	1 (100%)	1.000	-
Number of people sharing the child's room^c^	3 or more	3 (42.9%)	4 (57.1%)	0.665	1.748 (0.23-11.88)
Indoor pet^d^	Present	10 (33.3%)	20 (66.7%)	0.743	1.21 (0.33-4.64)
Maternal smoking during pregnancy^c^	Present	5 (38.5%)	8 (61.55%)	0.733	1.498 (0.32-6.55)
Maternal smoking during the first year of the child's life^c^	Present	5 (35.7%)	9 (64.3%)	0.745	1.290 (0.28-5.47)
Maternal smoking^c^	Present	7 (50%)	7 (50%)	0.103	2.933 (0.692-12.76)
Household member smoking	Present	4 (25%)	12 (75%)	0.506	0.646 (0.13-2.73)
Current presence of mold^d^	Present	6 (28.6%)	15 (71.4%)	0.713	0.803 (0.20-3.02)
Presence of mold during the first year of the child's life^d^	Present	5 (35.7%)	9 (64.3%)	0.745	1.29 (0.28-5.47)
Current presence of fitted carpets^c^	Present	17 (31.5%)	37 (68.5%)	-	-
Presence of fitted carpets during the first year of the child's life^d^	Present	2 (66.7%)	1 (33.3%)	0.230	4.644 (0.23-290.8)
Current tiled floor^c^	Present	16 (37.2%)	27 (62.8%)	0.143	5.777 (0.70-273)
Presence of tiled floor during the first year of the child's life^c^	Present	14 (35%)	26 (65%)	0.507	1.951 (0.42-12.69)
Current hardwood floor^c^	Present	0	2 (100%)	1.000	-
Presence of bare floor in the child's bedroom during the first year of the child's life^c^	Present	1 (12.5%)	7 (87.5%)	0.411	0.273 (0.01-2.44)
Current bare floor in the child's bedroom^c^	Present	1 (11.1%)	8 (88.9%)	0.244	0.232 (0.01-2.00)
Use of foam pillow during the first year of the child's life^c^	Present	14 (35%)	26 (65%)	0.299	2.908 (0.52-30.67)
Current use of foam pillow^c^	Present	12 (34.3%)	23 (65.75)	0.547	1.451 (0.37-6.42)
Use of synthetic fiber pillow during the first year of the child's life^c^	Present	0	6 (100%)	-	-
Current use of synthetic fiber pillow^c^	Present	3 (25%)	9 (75%)	0.732	0.672 (0.10-3.29)
Use of feather pillow during the first year of the child's life^c^	Present	1 (33.3%)	2 (66.7%)	1.000	1.163 (0.02-14.55)
Current use of feather pillow^c^	Present	2 (40%)	3 (60%)	0.645	1.499 (0.11-14.55)
Use of wool blanket during the first year of the child's life^d^	Present	6 (19.4%)	25 (80.6%)	0.026	0.269 (0.08-0.88)
Current use of wool blanket^d^	Present	3 (20%)	12 (80%)	0.338	0.453 (0.07-2.09)
Use of cotton blanket during the first year of the child's life^d^	Present	4 (33.3%)	8 (66.7%)	1.000	1.113 (0.21-5.13)
Current use of cotton blanket^d^	Present	4 (28.6%)	10 (71.4%)	1.000	0.834 (0.16-3.64)
Living in a rural neighborhood during the first year of the child's life^d^	Present	2 (22.2%)	7 (77.8%)	0.703	0.578 (0.05-3.56)
Living in an urbanneighborhood during the first year of the child's life/currently^d^	Present	15 (33.3%)	30 (66.7%)	0.703	1.733 (0.28-19.11)
Positive prick test^c^		13 (76.5%)	23 (62.2%)	0.364	1.954 (0.473-9.884)

ISAAC, International Study of Asthma and Allergies in Childhood; OR, *Odds Ratio*; 95%CI, 95% confidence interval.Student's *t*-test.

a Data for variables with categorical distribution are represented as number of patients with variable/total number of individuals included in the group and percentage related to the variable analyzed within the possible groups.

b Fisher-Freeman-Halton exact test.

c Fisher's exact test.

d *χ* ^2^ test.

**Table 5:** Characterization of the population born weighing less than 1500g considering asthma severity.

Variables^a^	Severe asthma	Mild asthma	*p*-value	OR (95%CI)
Birth weight (g)^b^	1076.44±341.98; 1150 (420-1455)	1074.25±172.83; 1063 (805-1295)	0.987	
BPD (present)^c^	4 (66.7%)	2 (33.3%)	0.620	2.278 (0.21-35.67)

g, grams; OR, *Odds Ratio*; 95%CI, 95% confidence interval.

a Data for variables with categorical distribution are represented as number of patients with variable/total number of individuals included in the group and percentage related to the variable analyzed within the possible groups; the data with numerical distribution are presented as mean±standard deviation; median (minimum-maximum).

b Student's *t*-test.

c Fisher-Freeman-Halton exact test.

## Discussion

This study showed a high prevalence of asthma in VLBW infants at school age, regardless of the presence or absence of BPD and associated risk factors. Studies that aim to analyze the influence of lung diseases in early life and their repercussions in adolescence and adulthood have increased in recent decades. Many of them try to answer the question of whether the BPD and/or preterm birth are risk factors for asthma.^4^^,^ 11 ^,^ 21 ^,^ 26 As found in the present study, other authors have observed a higher prevalence of asthma in VLBW infants when compared with the prevalence of asthma in the world population,^7^^,^ 11 ^,^ 19 ^,^ 22 ^,^ 27 with values for extremely premature infants ranging between 26%^5^^,^ 19 ^,^ 27 and 40%.^22^

Infants who develop BPD have a high prevalence of inflammation and decreased lung flow in childhood and adulthood.^1^^,^ 4 ^,^ 28 The initial inflammatory manifestations result from genetic susceptibility, endocrine disorders, infections, volutrauma, barotrauma, oxygen toxicity, and patent ductus arteriosus.^3^ The complexity of the presence and interaction of these factors, which contribute to the development and severity of BPD, can affect growth and lung function to a greater or lesser extent.^3^^,^ 4 In contrast, the influence of prematurity, BDP, or the interaction of both in the prevalence of asthma remains controversial.^11^^,^ 21 ^,^ 26 While most studies show a positive association between BPD^2^^,^ 7 ^-^ 14 ^,^ 17 ^,^ 20 ^,^ 22 ^,^ 26 or prematurity^28^^,^ 29 as a risk factor for asthma, one study did not observe this association.^21^ The present study observed a high prevalence of asthma; however, it was similar in VLBW infants who developed BPD and those who did not. Possibly, prematurity was a more important factor than BPD for the development of asthma in schoolchildren in the present sample, a fact confirmed by Bronstron et al.^9^ In addition to the present study, only one study failed to observe an association between BPD and asthma. Narang et al. studied 110 patients at 21 years, 60 full-term infants and 50 VLBW infants, and seven had BPD. These authors found no difference among VLBW infants with and without BPD regarding asthma.^21^

Another interesting finding is the influence of sex in the prevalence of obstructive lung diseases in children and adolescents. While male infants have narrower airways and increased prevalence of wheezing,^6^ the differences in lung function decrease with age and reverse in adolescence.^2^ It is believed that this occurs due to hormonal changes in women, with an increased risk of asthma at this age group.^2^ For the VLBW infants in the present study, there was no difference between sexes for both BPD and for asthma. This result was similar to that obtained by Fawke et al.,^14^ who studied respiratory symptoms and lung function in VLBW infants at 11 years and found no differences between the sexes. This explains the reduction of lung function differences between the sexes observed with age.

Most authors who have studied the association between prematurity/BPD and future asthma^5^^,^ 7 ^,^ 8 ^,^ 11 ^,^ 14 ^,^ 15 ^,^ 18 ^,^ 19 ^,^ 27 included the ISAAC^23^ risk factors and the Asthma Predictive Index (API).^6^ Although the present study did not specifically evaluate the API, all variables contained in that index, except for the number of eosinophils in peripheral blood, were analyzed, and no differences between both groups were observed. In the present study, the high prevalence of smoking in parents (25%), attendance at daycare in the first year of life (50%), low prevalence of breastfeeding, and history of maternal/paternal asthma in 20% of schoolchildren were noteworthy. Although the prevalence of asthma was high, the only risk factor associated with the CIQ was the use of wool blankets in the first year of life (*p*=0.026). This may reflect a higher exposure to allergens factors, which, together with inflammatory alterations caused by prematurity, could facilitate the onset of asthma due to early allergic sensitization.^6^ Unlike the present findings, Palta et al. observed a higher prevalence of asthma in VLBW infants with BPD when compared with those without BPD. In that study, the BPD group had a higher frequency of risk factors for asthma, which included repeated respiratory infections in the first year of life, family history of asthma, smoking parents, and family income.^7^ Kwinta et al. assessed risk factors for asthma in VLBW infants with or without BPD compared to healthy term infants. Those authors found that VLBW infants had more recurrent wheezing (OR=5.38, 95%CI 2.14-13.8) and a higher prevalence of risk factors, such as hospitalization and wheezing below 24 months.^11^

Risk factors for asthma may have different weights in children and adolescents who were VLBW or term newborn. While these factors have been thoroughly studied^6^ in children healthy at birth, the number of studies on VLBW infants is lower. Thus, the assessment of large cohorts following-up VLBW infants with and without BPD may clarify whether there are differences between them regarding the exposure to risk factors and development of future chronic diseases.

Numerous researchers have aimed to determine whether diseases in the neonatal period affect lung function in childhood, adolescence, and in later ages.^2^^,^ 4 ^,^ 5 ^,^ 7 ^-^ 15 ^,^ 17 ^-^ 22 ^,^ 27 While the relationship between BPD and asthma remains controversial,^4^^,^ 15 decreased lung function in VLBW infants throughout life has been documented.^8^^-^ 10 ^,^ 26 ^,^ 28 Some studies,^8^^,^ 19 ^,^ 20 ^,^ 26 including the present, showed lower lung volumes in the peripheral airways in children with asthma who were VLBW infants. The presence or absence of BPD did not increase the prevalence of asthma or airway obstruction in the present patients. Thus, prematurity may have a higher probability of association with asthma than BPD.

Gough et al. evaluated adults aged 24-25 years who had been VLBW infants with and without BPD. Those with BPD had twice as many episodes of wheezing and used three times more asthma medications.^22^ A prospective study showed that, at age 11, VLBW infants with BPD had lower lung volumes in spirometry and greater response to bronchodilators when compared with those who did not develop BPD.^14^ Doyle et al. followed-up a cohort of 147 VLBW infants with and without BPD, assessed at ages 2, 5, 8, 11, 14, and 18 years. These authors observed that lung volumes showed greater reductions between the age range of 8-18 years, more pronounced in the BPD group.^8^ Recently, von Hove et al. assessed the respiratory symptoms and lung function in adolescents who were VLBW infants, 28 with and 28 without BPD. They concluded that those with BPD were more likely to present abnormalities in lung function.^12^

The main cause of recurrent wheezing in children is atopic asthma. The present study failed to demonstrate an association of allergy (proven by clinical history and SPT) with BPD or asthma. Several studies, including the review by Perez and Navarro, evaluated the associations between preterm birth with and without BPD and future atopy/allergies, demonstrating that BPD has no longitudinal relationship with atopy or BHR.^2^^,^ 3 ^,^ 9 ^,^ 13 ^,^ 30 One study found an association between BPD and future atopy.^16^ Subsequently, several authors have failed to observe this association in children who were VLBW infants.^9^^,^ 13 ^,^ 30

One of the limitations of the present study and of that by Narang et al. was the small number of individuals included, compared to the total number of those who were eligible. Among the eligible subjects in the present study, 25 (30.86%) of those aged between 7 and 12 years refused to participate. In contrast, the present study assessed the risk factors for childhood asthma, which was not studied by Narang et al. Another aspect is that the present patients used a surfactant, while the subjects of Narang et al.^21^ did not. Moreover, it is worth mentioning as a limitation of the present study the impossibility of obtaining data on BPD severity, duration of mechanical ventilation, and other respiratory diagnoses in the neonatal period. A difficult-to-assess aspect that may constitute sample bias is whether the individuals who decided to participate in the present study had greater morbidities, including asthma, considering that VLBW infants with BPD had lower birth weight and were younger at time of examination.

Considering the diagnosis of asthma according to the ISAAC questionnaire, this study showed that VLBW infants, especially those of lower weight, had a higher prevalence of asthma, with no difference regarding the presence of BPD. Additional research including evolutionary comparison between infants, preschoolers, and schoolchildren through lung function by spirometry, lung clearance index, and bronchoalveolar lavage may bring light to the differences between the presence of BHR and asthma after prematurity and BPD. As these tools are difficult to use in clinical practice, multi-center epidemiological follow-up studies should be encouraged to allow for the establishment of samples that are adequate for comparisons in univariate and multivariate analyses.

The authors conclude that preterm infants with and without BPD have higher prevalence of asthma in childhood and adolescence when compared with the data reported in the literature for individuals in the same age group. The impact of BPD in the prevalence and severity of asthma remains controversial.
